# Accuracy of Computer-Aided Detection of Occupational Lung Disease: Silicosis and Pulmonary Tuberculosis in Ex-Miners from the South African Gold Mines

**DOI:** 10.3390/ijerph191912402

**Published:** 2022-09-29

**Authors:** Rodney Ehrlich, Stephen Barker, Jim te Water Naude, David Rees, Barry Kistnasamy, Julian Naidoo, Annalee Yassi

**Affiliations:** 1 Division of Occupational Medicine, School of Public Health and Family Medicine, University of Cape Town, Cape Town 7925, South Africa; 2 School of Population and Public Health, University of British Columbia, Vancouver, BC V6T 1Z3, Canada; 3 Diagnostic Medicine, Cape Town 7708, South Africa; 4 School of Public Health, University of the Witwatersrand, Johannesburg 2193, South Africa; 5 Office of the Compensation Commissioner for Occupational Diseases, Johannesburg 2001, South Africa; 6 Faculty of Health Sciences, University of the Witwatersrand, Johannesburg 2193, South Africa

**Keywords:** computer-aided detection, CAD, silicosis, tuberculosis, mining, South Africa

## Abstract

Background: Computer-aided detection (CAD) of pulmonary tuberculosis (TB) and silicosis among ex-miners from the South African gold mines has the potential to ease the backlog of lung examinations in clinical screening and medical adjudication for miners’ compensation. This study aimed to determine whether CAD systems developed to date primarily for TB were able to identify TB (without distinction between prior and active disease) and silicosis (or “other abnormality”) in this population. Methods: A total of 501 chest X-rays (CXRs) from a screening programme were submitted to two commercial CAD systems for detection of “any abnormality”, TB (any) and silicosis. The outcomes were tested against the readings of occupational medicine specialists with experience in reading miners’ CXRs. Accuracy of CAD against the readers was calculated as the area under the curve (AUC) of the receiver operating characteristic (ROC) curve. Sensitivity and specificity were derived using a threshold requiring at least 90% sensitivity. Results: One system was able to detect silicosis and/or TB with high AUCs (>0.85) against both readers, and specificity > 70% in most of the comparisons. The other system was able to detect “any abnormality” and TB with high AUCs, but with specificity < 70%. Conclusion: CAD systems have the potential to come close to expert readers in the identification of TB and silicosis in this population. The findings underscore the need for CAD systems to be developed and validated in specific use-case settings.

## 1. Introduction

Failure to deal with the regional health crisis of tuberculosis (TB) and silicosis among former miners in the post-apartheid era has left hundreds of thousands of such ex-miners with limited access to medical assessment, treatment, and related financial compensation [1,2,3]. Barriers are especially high for migrant workers and their families.

Governments, the private sector and civil society groups in the southern Africa region have recognized the need to reduce the burden of TB in the mining sector, as well as to promote control of silica exposure and silicosis as underlying drivers of TB [4,5,6]. In addition, inadequate access to social security has stirred governmental reform efforts and civil litigation aimed at securing compensation for those affected by mining lung disease. A number of regional cross-country programmes involving mass screening of former miners for TB and silicosis have consequently been undertaken. These include the Global Fund supported programme, TB in the Mining Sector in Southern Africa (TIMS) [7]; an outreach programme of the statutory Medical Bureau for Occupational Diseases (MBOD) [8]; and the programme of the Tshiamiso Trust, established following the settlement of a class action suit for silicosis and TB on behalf of miners and former miners from six South African gold mining companies [9,10].

The background population of concern in this study consists of former gold miners, who have not been regularly screened and who have limited access to health services. They are currently the target of the above-mentioned programmes, entailing that they undergo a medical examination to determine if they are eligible for workers’ compensation for occupational lung disease via the statutory system, recent civil settlement or both. In the South African mining industry, TB on its own is compensable for wage loss if active disease is diagnosed during employment or within 12 months thereafter. The legislation also covers permanent lung impairment, due to TB whenever assessed, if it can be attributed to active disease diagnosed during this qualifying period. Silicosis is compensable whenever diagnosed, as is the finding of a combination of silicosis and TB (silicotuberculosis) whether prior or active. Silicotuberculosis, earns a higher degree of compensation than silicosis alone. As a result of these compensation rules, the identification of prior, currently inactive TB (“prior” in this paper) is of great practical importance to miners and ex-miners from the South African mines.

In many cases, given the cost and distance facing many miners in rural areas in accessing clinical sites, there is pressure to complete at one visit the screening for silicosis and TB as compensable diseases, as well as for active TB requiring treatment. In conjunction with a medical and symptom history, plain chest radiography is essential to these programmes. However, a major bottleneck is the shortage of expert medical personnel to provide timely assessment of chest X-rays (CXRs) for features of TB and silicosis. In recent years, the World Health Organization (WHO) has included in its recommendations for early detection of active TB the use of plain chest radiography for screening and triage [11]. For a “community-based triage or referral test”, the recommendation is a low cost per test, and a minimum sensitivity of 90% and specificity of 70% [12]. Current and former miners and other workers with silica exposure are one of the populations identified by WHO for systematic screening for TB disease [13].

Simultaneously, advances have been made in using computer-aided detection (CAD) for TB [14,15,16,17]. Commercially available systems have achieved promising metrics of agreement [area under the curve (AUC)] of the receiver operator characteristic (ROC) curve when compared to microbiological reference standards or human expert readers. CAD is now conditionally recommended by WHO for detection of active TB disease in adult populations “in place of human readers for interpreting digital chest X-rays for screening and triage”, although with a “low certainty of evidence” [13]. CAD systems have also been developed for pneumoconiosis, including silicosis, although with a much smaller field validation literature than for TB [18,19].

CAD offers the possibility of high volume, sensitive radiological screening for both TB and silicosis at low relative cost in working and former miners. However, the gold mining population in particular is characterised by a high prevalence of silicosis, radiological changes attributable to active and/or prior TB, and HIV infection [20,21]. This combination greatly complicates the radiological identification of TB and silicosis. As a fibrotic disease with a nodular appearance on the CXR, or in some cases areas of massive fibrosis, silicosis may mimic or mask TB changes [22,23,24]. Conversely, TB may present features mimicking silicosis, such as bilateral nodulation or massive fibrosis; or silicosis may be obscured by distorted lung architecture, volume loss or extensive pleural thickening. It has also been suggested that the presence of silica particles in the lung promotes a predominantly fibroblastic tuberculous reaction and a nodular appearance similar to that of silicosis [22,25].

The application of CAD in this setting therefore faces singular difficulties not found in other TB screening settings. Recent WHO guidelines recognise the necessity of testing or calibrating CAD systems for local settings [13,26]. Only one previous validation of CAD in the setting of this study has been published—using mainly preselected chest images from a compensation archive, and the decisions of the statutory compensation panel (the MBOD) as the reference standard [27]. Three of the four proprietary CAD systems tested achieved a “high” degree of accuracy (AUC > 0.85) [28] in distinguishing abnormal CXRs from normal, and specifically those with features of silicosis and/or TB (without distinction between prior or active disease) from normal. However, when a distinction was required between TB and silicosis, or between TB alone and silicosis plus TB, agreement deteriorated, with AUCs ranging from poor to moderate (~0.65–0.81). This indicated that further work was needed in the development of CAD for use in this population.

The objective of the current study was to assess the ability of CAD software systems to detect TB and, separately, silicosis on the CXRs of former gold miners. The current study differed in two ways from our previous one: (a) CAD outcomes were compared with the readings of expert readers using the International Labour Organization (ILO) Classification [29] rather than relying on medicolegal readings performed for compensation purposes; and (b) the images were taken from field screening of consecutive former miners rather than relying on images that were preselected from an archive.

## 2. Materials and Methods

### 2.1. Population and Sampling

The study was conducted according to STARD guidelines for diagnostic accuracy [30]. The target group consisted of former gold miners who had worked in the Klerksdorp gold mining area of South Africa, had more than ten years of service and had held a dusty occupation. The sample comprised those who presented themselves at the examination points on the screening days and whose claims under South African miners’ compensation statute were forwarded to the MBOD in Johannesburg. Examinations were conducted over four days at each of three rural South African sites between November 2019 and February 2020 and yielded a sample of 501: Stilfontein, Gauteng province (n = 51); and Alice and Bizana, Eastern Cape province (n = 197 and 253, respectively). No selection occurred as all participants had their CXRs read.

Information on age was extracted from metadata contained within the digital CXR. Information on length of service was obtained for those ex-miners (n = 257) who by the time of the study had had their claims matched with their service record and adjudicated by the MBOD, which requires such proof of service. It transpired that six participants had two images submitted—these were retained in the sample read.

The CXRs therefore represented a field sample of miners with an occupational history that carried an elevated risk of silicosis, silicotuberculosis, and/or permanent impairment due to TB alone, and for whom the incentive of compensation existed. The outcomes of interest in this study were TB and silicosis. Definitions are given below. CXRs were taken with a Delft Easy DR system incorporating a Canon direct digital flat panel detector.

While our previous CAD study had sufficient power to calculate the relevant metrics of agreement (AUC, sensitivity and specificity) with acceptable precision using a sample of 330 plain CXR images [27], the present study had a larger sample to allow for the possibility of greater random variation in CXR quality and reading accuracy attributable to this being a field study rather than a study conducted on a pre-selected sample of CXRs. With a conservative expected silicosis prevalence (based on a 2008 study of ex-miners [20]) of 20% or greater in our sample, the available sample of 501 provided sufficient power at 82.6% (with a 95% significance level) to show an improvement in AUC in the detection of silicosis, specifically from 0.730 (derived from our previous study [27]) to 0.850 (high accuracy. The expected prevalence of radiological changes attributable to TB was 30% [20].

### 2.2. Reading of CXRs

Anonymised chest images were classified by three occupational medicine specialists with extensive experience in interpreting CXRs from a gold mining population (“reader 1” = author DR; “reader 2” = consensus during the read between authors RE and JtWN). Readers had no other information about participants. The accuracy of CAD was analysed separately for reader 1 and reader 2 and both sets of results are presented. The CXR reading form adapted for this purpose covered image quality, silicosis as present or absent, and if present, the International Labour Organization (ILO) Classification [29] reading for nodular profusion and/or progressive massive fibrosis (PMF) (Figure S1, supplementary material). The profusion score in the Classification refers to the density of nodulation on the CXR on a major ordinal scale from zero (no nodulation) to three (very dense nodulation). These categories are represented by standard consensus images provided as part of the Classification. In addition, there is a minor scale in which a second number can be added by the reader to the primary assessment to allow for intermediate values, e.g., 0/0, 0/1, 1/0, 1/1, 1/2, etc. In this study, 1/0 and 1/1 were used as the thresholds for the presence of silicosis as identified by the readers, representing “borderline present” and “definitely present”, respectively. Separate analyses were carried out for images read as ≥1/0 and ≥1/1 to examine the effect on CAD accuracy of excluding the borderline category 1/0.

For TB, the reading form required judgement on the presence and extent of a variety of individual radiological features of TB associated with prior or active TB (Figure S1, supplementary material). An ordinal scale was used for classification of likelihood of current active TB (none, possible, probable/definite), and the same scale for prior TB. The presence of features of “other“ disease including COPD, cardiovascular abnormality, pleural abnormality, etc., was also recorded. The analysis in this study, which did not include bacteriological tests of TB, is that of radiological abnormality identified by CAD and/or the external readers as consistent with TB, without distinguishing between active or prior disease. The definition of TB also included both “possible” and “probable/definite” disease as read by the external readers. The effect on CAD accuracy of narrowing this definition to “probable/definite” disease was examined separately.

The chest images were submitted to the two CAD vendors whose systems are designated systems A and B. Both had achieved relatively high accuracy in our earlier study [27]. System A was provided by Delft Imaging: CAD4TB, version 7, for reading for TB (https://www.ai4hlth.org/product-profiles/Delft-Imaging, accessed on 24 August 2021) [31]; and CAD4Silicosis, Version 1.0.0, for silicosis, using the foundation from CAD4TB and an ensemble of networks. System B was provided by EPCON SA: XrayAME, Version 1, for reading TB and “any abnormality” (https://www.ai4hlth.org/product-profiles/EPCON, accessed on 9 September 2021). The latter category included but did not separately identify silicosis. The outputs of the two systems are summarised in Table 1. None of the study images were used for training of the CAD systems.

### 2.3. Statistical Analysis

All statistical analysis was performed using R Statistical Software version 4.1.2, with the pROC package (version 1.18.0, Xavier Robin et al., Geneva, Switzerland) and the irr package (version 0.84.1, Matthias Gamer et al., Hamburg, Germany). For comparison of inter-reader agreement across binary categorical variables, Cohen’s kappa was calculated. ILO quality grade was dichotomised as 1 (“Good”) or 2 (“No technical defect likely to impair classification of the radiograph for pneumoconiosis”) vs. 3 (“Acceptable, with some technical defect but still adequate for classification purposes”) or 4 (“Unreadable”). The descriptive terms of extent of agreement were: ≤0, poor; 0.01–0.20, slight; 0.21–0.40, fair; 0.41–0.60, moderate; 0.61–0.80, substantial; and 0.81–1.00, almost perfect agreement [32].

To assess the ability of the CAD systems to identify the outcomes of interest, ROC analysis was used to calculate the AUC as the primary metric of agreement between different CAD systems and the external readers. An AUC > 0.85 was regarded as “high”, between 0.75 and 0.85 as “moderate”, and below 0.75 as “poor” [28]. Additionally, sensitivity and specificity were calculated from the ROC curves using two threshold criteria. The first was Youden’s index which provides the point on the ROC curve at which the combination of sensitivity and specificity is maximized [sensitivity + specificity − 1]. The alternative was choosing a contextually relevant value for sensitivity—90% in this case (drawing on the WHO “minimal” benchmark for screening for active TB [12]—and allowing specificity to find its related value. AUCs were compared statistically using the DeLong test [33].

Finally, the influence of, age and length of mining service on agreement between CAD and the external readings was explored using logistic regression modelling and estimation of interaction terms. Analysis using length of service was restricted to the 257 participants for whom this information was confirmed.

## 3. Results

### 3.1. Outcome Frequencies

Tables S1–S4 (supplementary material) set out the proportion of each outcome or category read, respectively by the two readers. Among abnormalities other than silicosis or TB reflected in Table S1, categories with the highest frequency were hyperinflation, bullae, other COPD related signs; cardiomegaly, ectatic aorta and other cardiovascular abnormalities; and pleural abnormality (including costophrenic angle blunting) not believed to be TB related.

### 3.2. Inter-Reader Agreement

A sample of 501 CXR images was read. The percentage agreement on ILO quality grade 1 or 2 vs. grades 3 or 4 was 98.6 percent. There were too few images classified as grade 3 or 4 (7, reader 1; 10, reader 2) to calculate kappa.

The inter-reader agreement on the various outcomes as expressed by kappa is summarized in Table 2. (See also Tables S5–S11, Supplementary Material). There was substantial agreement between readers on the presence of TB including both “possible” and “probable/definite” disease, but only moderate agreement when the definition was restricted to “probable/definite” TB. Agreement was moderate on the presence of “any abnormality” and silicosis (however defined), and fair for silicotuberculosis (however defined).

### 3.3. Comparative Performance of the CAD Systems against External Readers

#### 3.3.1. Any Abnormality

**Figure 1:**
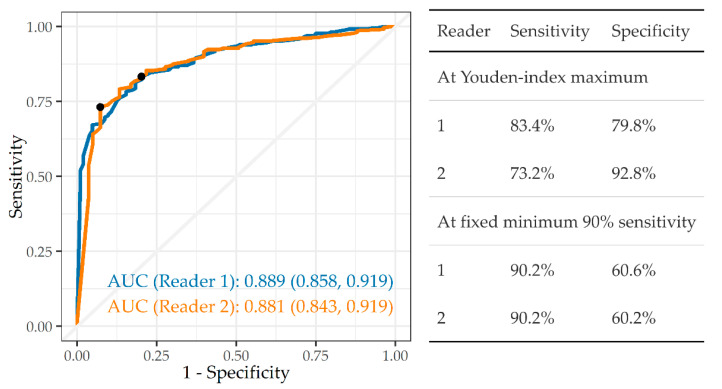
ROC curve, AUC, and sensitivity and specificity of system B in detecting “any abnormality”, against readers 1 and 2 (n = 501).

Figure 1 indicates that system B was able to distinguish “any abnormality” from normal with a high degree of accuracy against both readers 1 and 2 (AUC 0.889, 0.881, respectively). The tables embedded in the figures provide the sensitivity and specificity at two different criteria—Youden’s Index and 90% sensitivity. Using Youden’s Index, sensitivity and specificity have similar values at 83.4% and 79.8% against reader 1, respectively. By contrast, they are somewhat unbalanced at 73.2% and 92.8%, respectively against reader 2. On the 90% sensitivity criterion, specificity falls quite sharply to 60.6% and 60.2% against readers 1 and 2, respectively.

#### 3.3.2. Tuberculosis

**Figure 2:**
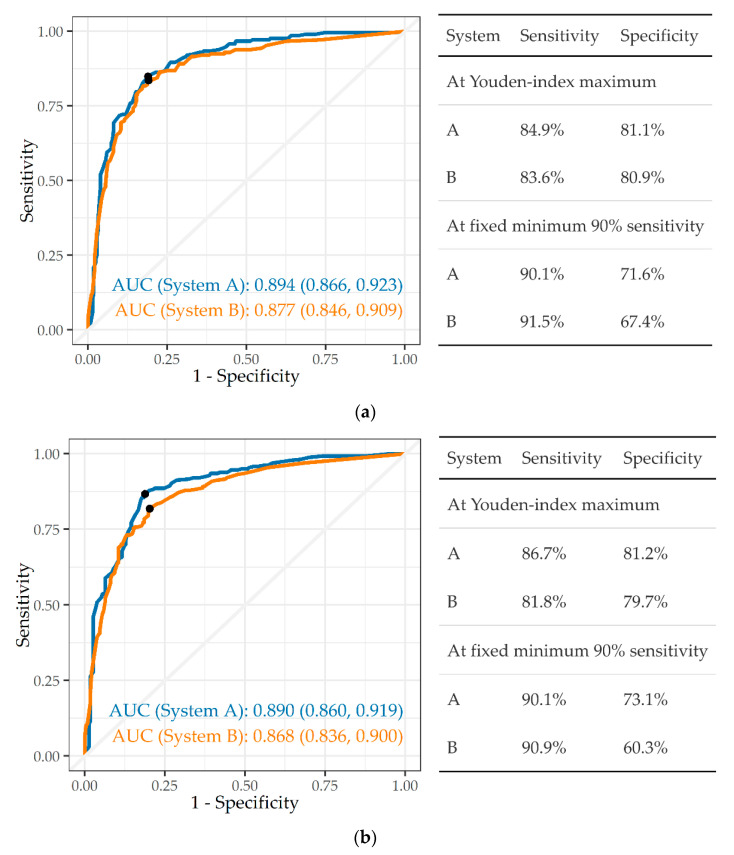
(**a**) ROC, AUC, and sensitivity and specificity of two CAD systems in detecting tuberculosis, against reader 1 (n = 501). (**b**) ROC, AUC, and sensitivity and specificity of two CAD systems in detecting tuberculosis, against reader 2 (n = 501).

The ability to identify abnormalities consistent with TB (whether active, prior or both) was examined for both CAD systems A and B (Figure 2a,b). Both achieved high agreement (AUC > 0.85) against both readers. The specificity of system A at the 90% sensitivity criterion was 71.6% against reader 1, and that of system B only slightly lower at 67.4% (*p* = 0.17 for difference from that of reader 1). Against reader 2, the specificity of system A was 73.1%, but that of system B markedly lower at 60.3% (*p* < 0.001).

The effect of narrowing the TB analysis to “probable/definite” disease, i.e., excluding the “possible” category, is presented in Table S12 (supplementary material). There were no significant differences in the AUC between the two definitions.

#### 3.3.3. Silicosis and Silicotuberculosis

**Figure 3:**
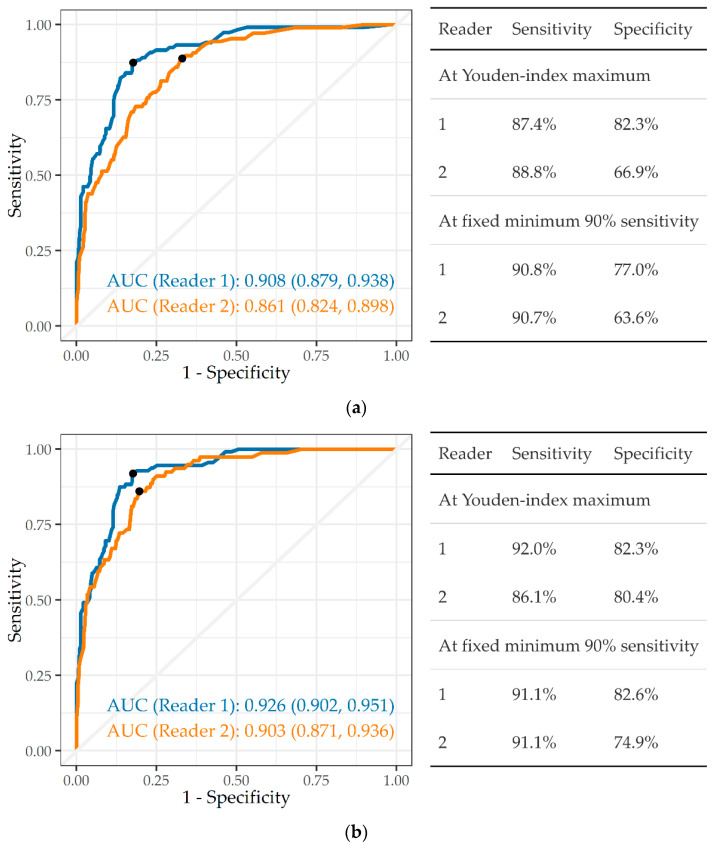
(**a**) ROC curve, AUC, and sensitivity and specificity of a single CAD system (A) in detecting silicosis ILO profusion ≥ 1/0, against readers 1 and 2 (n = 501). (**b**) ROC curve, AUC, and sensitivity and specificity of a single CAD system (A) in detecting silicosis ILO profusion ≥ 1/1, against readers 1 and 2 (n = 501).

System A was the only one to provide a score specifically for silicosis. Figure 3a,b therefore compare readers, not systems. First, CXRs classified by the external readers as ILO profusion ≥ 1/0 were defined as silicosis, whether TB was present or not, and all other images as “no silicosis” (Figure 3a). CAD system A achieved a high AUC of 0.908 and 0.861 against reader 1 and reader 2, respectively. At the 90% sensitivity criterion, this translated into a specificity of 77.0% against reader 1, and 63.6% against reader 2.

When silicosis was classified as ILO ≥ 1/1 (Figure 3b), there was an improvement in the AUC against both reader 1 (0.908 to 0.926, *p* = 0.36) and against reader 2 (0.861 to 0.903, *p* = 0.091). Accordingly, on the 90% sensitivity criterion, specificity improved, particularly against reader 2.

**Figure 4:**
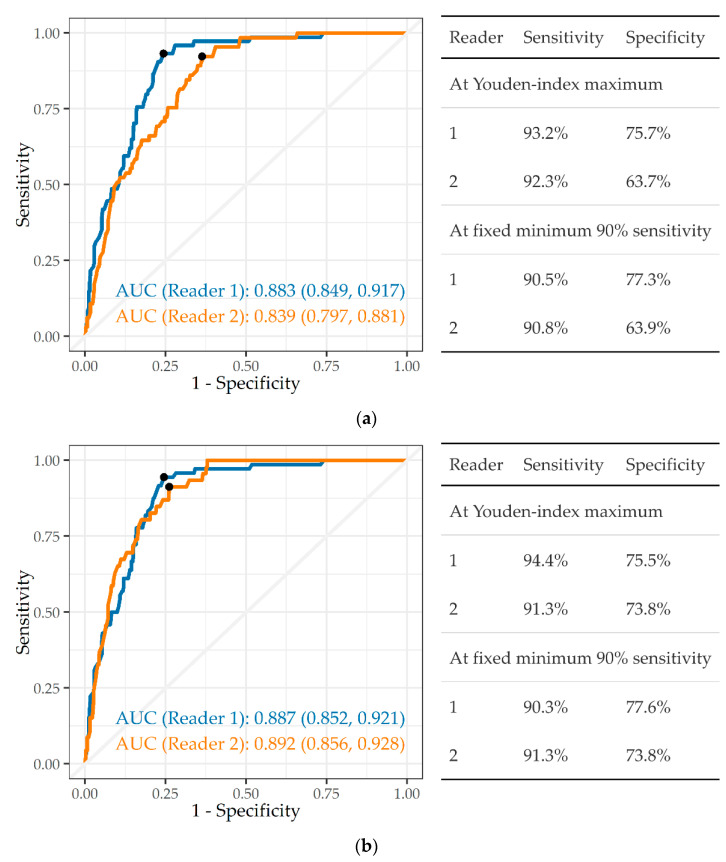
(**a**) ROC curve, AUC, and sensitivity and specificity of a single CAD system (A) in detecting silicotuberculosis, ILO silicosis profusion ≥ 1/0, against readers 1 and 2 (n = 501). (**b**) ROC curve, AUC, and sensitivity and specificity of a single CAD system (A) in detecting silicotuberculosis, ILO silicosis profusion ≥ 1/1, against readers 1 and 2 (n = 501).

Silicotuberculosis was defined as a CXR showing both silicosis and TB. The AUCs were high and moderate against readers 1 and 2, respectively (0.883, 0.839) when using profusion ≥ 1/0 for the silicosis component (Figure 4a). For silicosis ≥ 1/1 (Figure 4b) the AUCs were both high (0.887, 0.892). As with silicosis alone, specificity was lower against reader 2 than against reader 1.

### 3.4. Variation in AUC by Covariates

Age ranged from 32 to 92 years [median 62 years, interquartile range (IQR) 58–66]. The number of years worked in mining was available for only a subset of 257 miners, ranging from 0.9 to 45 years (median 21.1 years, IQR 14.0–28.6). In logistic regression analysis, there was no interaction between age and CAD, nor between length of service and CAD in the prediction of the external readings (Table S13, supplementary material). Further, inclusion of age, service or an interaction term did not significantly improve the AUC over the use of CAD outcome alone for either of the readers.

## 4. Discussion

The purpose of the study was to determine whether CAD systems for TB and silicosis could reach acceptable performance standards in a former gold miner population with a very high prevalence of mixed radiological abnormality and where which much of the TB fell into the category of non-active radiological disease. Of secondary interest was the effect on CAD performance of moving from a highly pre-selected sample in a previous study [27] to a field screening sample.

### 4.1. Comparison with Previous CAD Study

This follow-up study represented a more testing validation design than our previous study [27] for a number of reasons. The reference reading for 86% of the CXRs used in the original study was that of the Certification Committee under South Africa’s Occupational Diseases and Mines and Works Act (ODMWA). In the current study, the reference standard was the clinical readings of external readers. While the previous study used preselected groups of CXR images, this study used CXRs from field screening of ex-gold miners, i.e., taken in real world conditions. This approach is in accordance with the recommendation made by WHO and other commentators that different reference standards and validation sets be used in test trials [13,15]. Preselection of the images in the previous study would have favoured higher quality images with clearer-cut classifications than consecutive images from a field study. The medicolegal threshold used to define silicosis in the prior study required an ILO major scale profusion of >2 in the absence of lung function tests or TB, whereas this study used 1/0 and 1/1 as thresholds. Lastly, the Certification Committee members had information available to them on TB history, current TB investigations and duration of service. This may have reduced the number of “grey area” cases in which TB was misclassified as silicosis in the reference set.

Compared to the previous study, System A was approximately able to replicate its performance in detection of TB, and separately, in identification of silicosis. System B substantially improved its performance in detecting TB and maintained its performance for detecting “any abnormality”.

### 4.2. Performance in Relation to WHO TB Screening Guideline

Our previous study [27] used the Youden’s index criterion as the point on the curve which maximises the sum of the two metrics. This may not be suitable for settings which place more or less weight on sensitivity or specificity, respectively. In the current study, we therefore chose a threshold with a relatively high sensitivity of 90% (drawing on the WHO “minimal” recommendation for active TB screening of 90% sensitivity and 70% specificity) [12] and used specificity as the variable index of performance. Application of this criterion to “any abnormality” and silicosis was therefore for illustrative purposes. Costs were not considered.

System A was able to achieve the “90/70” threshold for both silicosis and TB against one, although not both readers. System B did not meet this threshold for TB, although its specificity against reader 1 was not far short of the 70% mark. It also attained relatively low specificities for “any abnormality” against both readers; this may be at least partly due to heterogeneity of this omnibus category which includes lung hyperinflation, cardiomegaly, non-TB related pleural abnormality and other infection, among others. This highlights the current inability of CAD systems developed for TB to flag non-TB abnormalities which may be clinically important, as noted elsewhere [13,17].

If one were to accept a lower sensitivity, higher specificities could be attained. This is reflected in the results using the Youden’s Index. This is particularly so for system B in respect of TB (see Figure 2a,b) where acceptance of sensitivities of 82.8% and 81.9% for the two readers would result in specificities of 82.5% and 80.1%, respectively. In practice, users of screening radiology have to decide on the desired trade-off [26]. In this setting, where finding all the cases of silicosis or TB (i.e., minimising false negatives) is important, there is likely to be premium on sensitivity. Such a system would identify most CXRs which are normal or which have a very low likelihood of the disease of interest. This would yield considerable savings in time and effort by concentrating on abnormal CXRs for further diagnosis or compensation assessment. However, this approach would come at the cost of a relatively high proportion of false positives. The optimal balance would have to take both sets of costs into account.

Further training of the CAD systems on CXRs from the target population would be expected to improve performance metrics over time. Additionally, as evidenced in this study, higher AUCs were attained by using the more specific definition of silicosis as read by the reference readers, namely ILO ≥ 1/1 rather than ≥1/0. Accuracy might also be improved by varying the definition of silicosis according to the individual’s cumulative exposure to silica dust, using a lower ILO threshold for individuals with higher exposure [34]. However, information on cumulative silica exposure was not available for this study.

### 4.3. Limitations and Strengths

This is, to our knowledge, the first field study of CAD focused on silicosis and using consecutive CXRs from an identified use-case setting. Following the earlier study by our group [27], this is also to our knowledge only the second study to focus on silicosis and radiological TB in a population with a high proportion of prior TB. Training images used by the CAD vendors were drawn mostly from the population (compensation examinations for ex-goldminers) on which the CAD was tested. This enabled the study sample to replicate the prevalence of disease expected in the use-case setting, a general principle recommended in the literature [13,16,26]. The quality of the chest images was high, as would be expected with digital radiography.

The study has a number of limitations. The reference standard for silicosis and for prior TB was of necessity that of human readers. The highest inter-reader agreement (“substantial”) was for TB (any form). Reader agreement on the presence of silicosis, was only “moderate” (kappa = 0.58 for ILO ≥ 1/1). This figure fell at the lower end of the range of two-rater weighted kappas for silicosis from a previous study of miners in South Africa, (0.58 to 0.73) [20], but well above the multi-rater kappa of 0.37 for “parenchymal abnormalities” in a more recent study [35]. The disagreement between expert readers, varying by outcome, reflects the difficulties of reading CXRs from a population with a high prevalence of both silicosis and TB, especially where the readers have no other information on the individual miner. AUCs consequently varied between readers, with a trend to lower agreement of the CAD systems with reader 2. “Reader 2” represented a consensus of two readers, which might impair comparability with a single reader, although the direction of any effect is difficult to predict. Additionally, in the absence of a gold standard, one cannot conclude from the difference in AUCs that reader 1 is the more accurate reader of silicosis.

The study was not designed to test whether CAD was able to identify active TB as no information was available on symptoms, bacteriological testing, nor other TB risk factors such as HIV infection. The relevance of TB, whether active or prior, to the compensation process for mining lung disease in South Africa was described earlier. Research is needed on the utility of CAD in identifying active TB in a setting where there is a high proportion of prior TB and resultant permanent abnormalities on the CXR.

Finally, the findings may not be generalisable to other occupational settings. These include the active gold mining workforce in South Africa, who are on average younger than former miners, less likely to have chronic lung disease, are screened at least annually, and have access to mine medical services [36,37]. Similar considerations would apply to generalising to occupational and general populations elsewhere.

## 5. Conclusions

We have shown that two different CAD software systems have similar capability, relative to that of expert readers, of detecting radiological signs of silicosis and TB on CXRs taken in field screening conditions among former miners from the South African gold mines. The most accurate system is capable of meeting a benchmark standard of 90% sensitivity and 70% specificity for the two diseases of interest, while a second system is close enough to suggest that with further system development and training, it could demonstrate similar accuracy in detecting “any abnormality” and TB. The demonstration that at least one CAD system is capable of detecting silicosis in a population with a high background prevalence of prior TB is particularly noteworthy. The next stage is to use CAD in practice for this population, and to monitor and evaluate its impact on system efficiency [38], on satisfaction of clinicians and medical adjudicators, and on ethical practice [39].

## Figures and Tables

**Table 1:** Features and output definitions of CAD systems used in this study ^1^.

System	A	B
Any abnormality	-	Numerical: 0–100; Categorical: TB or other abnormalities (including silicosis) present vs. absent (CXR normal).
TB	Numerical: 0–100; Categorical: TB present vs. absent.	Numerical: 0–100; Categorical: TB present vs. absent.
Silicosis	Numerical: 0–100; Categorical: silicosis present vs. absent	-
Silicotuberculosis (calculated)	Numerical: 0–100 (Sum TB, silicosis scores)/2; Categorical: silicotuberculosis present vs. absent	-

^1.^ Cutpoint for categorical analysis chosen from receiver operating characteristic curve (see text).

**Table 2:** Agreement (kappa statistic) between external readers 1 and 2 ^1^.

	N1 ^2^	N2 ^3^	Kappa	95% CI	Agreement
Any abnormality	397	418	0.561	0.474, 0.648	Moderate
TB (possible or probable/definite) ^4^	213	264	0.655	0.570, 0.741	Substantial
TB probable/definite ^4^	128	216	0.572	0.491, 0.653	Moderate
Silicosis ≥ ILO 1/0 (irrespective of TB)	119	109	0.501	0.413, 0.588	Moderate
Silicosis ≥ 1/1 (irrespective of TB)	112	80	0.565	0.479, 0.650	Moderate
Silicotuberculosis ≥ 1/0	74	66	0.320	0.232, 0.407	Fair
Silicotuberculosis ≥ 1/1	72	47	0.384	0.299, 0.469	Fair

^1.^ “Reader 2” = consensus read between two readers. ^2.^ Number read as disease positive by reader 1. ^3.^ Number read as disease positive by reader 2. ^4.^ Whether read as active, prior or both.

## Data Availability

The anonymised datafile and key used in this study can be found at: https://doi.org/10.25828/sntw-6721 (accessed on 28 Mar 2022).

## Supplementary Materials

The following supporting information can be downloaded at: https://www.mdpi.com/article/10.3390/ijerph191912402/s1, Figure S1: CXR reading form; Table S1: Any abnormality—frequency by reader (N = 501); Table S2: Tuberculosis—frequency by reader (N = 501); Table S3: Silicosis—frequency by reader (N = 501); Table S4: Silicotuberculosis—frequency by reader (N = 501); Table S5: Any abnormality—agreement between readers (N = 501); Table S6: Tuberculosis (any vs. no TB)—agreement between readers (N = 501); Table S7: Tuberculosis (probable/definite vs. no TB/possible TB)—agreement between readers (N = 501); Table S8: Silicosis ≥ 1/0—agreement between readers (N = 501); Table S9: Silicosis ≥ 1/1–agreement between readers (N = 501); Table S10: Silicotuberculosis (ILO ≥ 1/0 plus TB)—agreement between readers (N = 501); Table S11: Silicotuberculosis (ILO ≥ 1/1 plus TB)—agreement between readers (N = 501); Table S12: Effect on AUC of alternative definitions of TB, readers 1 and 2; Table S13: Interaction between CAD output, age and length of service in predicting external reader classification of silicosis and silicotuberculosis (system A).
