# Supplementary material for: Accuracy of Computer-Aided Detection of Occupational Lung Disease: Silicosis and Pulmonary Tuberculosis in Ex-Miners from the South African Gold Mines
Source: Int J Environ Res Public Health. 2022 Sep 29;19(19):12402. doi: 10.3390/ijerph191912402 (PMC9565035; doi:10.3390/ijerph191912402)
Supplement: Supplementary file 1 [file ijerph-19-12402-s001.zip › ijerph-1862372-SI.pdf]

Supplementary material 1: Supplementary figures and tables

Frequency

Table S1. Any abnormality – frequency by reader (N = 501)

	Reader 1		Reader 2	
	N	Percent	N	Percent
No abnormality (normal)	104	20.8%	83	16.6%
Abnormality other than TB or silicosis	139	27.7%	111	22.2%
TB or silicosis[a]	258	51.5%	307	61.3%
Any abnormality	397	79.2%[b]	418	83.4%[b]
Total	501	100.0%	501	100.0%

[a] Silicosis ≥ 1/0, any TB, or both.
[b] $p = 0.105$ for difference

Table S2. Tuberculosis[a] - frequency by reader (N = 501)

	Reader 1		Reader 2	
	N	Percent	N	Percent
No TB	288	57.5%	237	47.3%
Possible TB	85	17.0%	48	9.6%
Probable/definite TB	128	25.5%	216	43.1%
TB (any)	213	42.5%[b]	264	52.7%[b]
Total	501	100.0%	501	100.0%

[a] Active, prior or both.
[b] $p = 0.002$ for difference.

Table S3. Silicosis – frequency by reader (N = 501)

	Reader 1		Reader 2	
	N	Percent	N	Percent
No silicosis (ILO 0/0 or 0/1)	382	76.2%	392	78.2%
Silicosis = 1/0	7	1.4%	29	5.8%
Silicosis ≥ 1/1	112	22.4%	80	16.0%
Silicosis ≥ 1/0	119	23.8%[a]	109	21.8%[a]
Total	501		501	

[a] p = 0.498 for difference

Table S4. Silicotuberculosis[a] – frequency by reader (N = 501)

	Reader 1		Reader 2	
	N	Percent	N	Percent
No silicotuberculosis (ILO 0/0 or 0/1)[b]	427	85.2%	435	86.8%
Silicotuberculosis = 1/0	2	0.4%	19	3.8%
Silicotuberculosis ≥ 1/1	72	14.4%	47	9.4%
Silicotuberculosis ≥ 1/0	74	14.8%[c]	66	13.2%[c]
Total	501		501	

[a] Both silicosis and tuberculosis.
[b] ILO profusion reading for the silicosis component.
[c] p = 0.524 for difference

Inter-reader agreement

Any abnormality

Table S5. Any abnormality[a] - agreement between readers (N = 501)

		Reader 2		
		Normal	Abnormal	Total
**Reader 1**	No abnormality	60	44	104
	Abnormality	23	374	397
Total		83	418	501
Kappa = 0.561 (95% CI 0.474, 0.648) – moderate agreement.				

Tuberculosis

Table S6. Tuberculosis[a] (any vs. no TB) - agreement between readers (N = 501)

		Reader 2		
		No TB	TB (any)	Total
**Reader 1**	No TB	219	69	288
	TB (any)	18	195	213
Total		237	264	501
Kappa = 0.655 (95% CI 0.570, 0.741) - substantial agreement.				

[a] Active, prior or both

Table S7. Tuberculosis[a] (probable/definite vs. no TB/possible TB) – agreement between readers (N = 501)

		Reader 2		
		No TB[a] or Possible TB[b]	Probable TB[c]	Total
**Reader 1**	No TB/ possible TB	279	94	373
	Probable/definite TB	6	122	128
**Total**		285	216	501
Kappa = 0.572 (95% CI 0.491, 0.653) - moderate agreement.				

[a] Active, prior or both

<u>**Silicosis and silicotuberculosis**</u>

Table S8. Silicosis ≥ 1/0 – agreement between readers (N = 501)

		**Reader 2**		
		No silicosis	Silicosis	Total
**Reader 1**	No silicosis	343	39	382
	Silicosis	49	70	119
Total		392	109	501
Kappa = 0.501 (95% CI 0.413, 0.588) - moderate agreement.				

Table S9. Silicosis ≥ 1/1 – agreement between readers (N = 501)

		**Reader 2**		
		No silicosis or ILO 1/0	Silicosis ≥ 1/1	Total
**Reader 1**	No silicosis or ILO 1/0	371	18	389
	Silicosis ≥ 1/1	50	62	112
Total		421	80	501
Kappa = 0.565 (95% CI 0.479, 0.650) - moderate agreement.<br><br>• No significant difference between for kappas for ILO ≥ 1/0 and ILO ≥ 1/1: 0.064 (95% CI -0.059, +0.18, p=0.312).				

Table S10. Silicotuberculosis (ILO ≥ 1/0[a] plus TB) – agreement between readers (N = 501).

		**Reader 2**		
		SilicoTB negative	SilicoTB positive	Total
**Reader 1**	SilicoTB negative	390	37	427
	SilicoTB positive	45	29	74
Total		435	66	501
Kappa = 0.320 (95% CI 0.232, 0.407) - fair agreement.				

[a] ILO profusion for reading of silicosis component.

Table S11. Silicotuberculosis (ILO ≥ 1/1[a] plus TB) – agreement between readers (N=501).

		**Reader 2**		
		SilicoTB negative	SilicoTB positive	Total
**Reader 1**	SilicoTB negative	409	20	429
	SilicoTB positive	45	27	72
Total		454	47	501

Kappa = 0.384 (95% CI 0.299, 0.469) - fair agreement.

- No significant difference between kappas for ILO $\geq$ 1/0 and ILO $\geq$ 1/1 for silicosis component: 0.064 (95% CI -0.058, +0.186; p=0.307)

[a] ILO profusion for reading of silicosis component.

CAD accuracy - alternative definitions of tuberculosis

Table S12. Effect on AUC of alternative definitions of TB , readers 1 and 2

**CAD System**	**External reader**	**Possible or probable/definite TB (AUC, 95% CI)**	**Probable/definite TB (AUC, 95% CI)**	**P (DeLong's test comparing ROC curves)**
A	1	0.894 (0.866, 0.923)	0.874 (0.841, 0.908)	0.38
	2	0.890 (0.860, 0.919)	0.886 (0.857, 0.915)	0.86
B	1	0.877 (0.846, 0.909)	0.874 (0.842, 0.906)	0.89
	2	0.868 (0.836, 0.900)	0.890 (0.861, 0.918)	0.31

AUC, area under the curve; CI confidence interval; ROC, receiver operating characteristic

Interaction

Table S13. Interaction between CAD output, age and length of service in predicting external reader classification of silicosis and silicotuberculosis (system A)[a]

Outcome	Reader	Logistic model	OR (95% CI) CAD (/10 points)	OR (95% CI) (i) age (/10 yr) (ii) age×CAD	OR (95% CI) (i) service (/10 yr) (ii) service×CAD	AUC	p[b]
Silicosis (ILO ≥ 1/0)	1	CAD only (N=497)	1.88 (1.69, 2.09)			0.908	-
		CAD, age (N=493)	1.88 (1.69, 2.09)	1.35 (0.63, 2.92)<br>0.99 (0.98, 1.01)		0.907	0.88
		CAD, service (N=254)	1.58 (1.37, 1.83)		1.26 (0.69, 2.29)<br>1.01 (0.99, 1.03)	0.893	0.14
		CAD, age, service (N=252)	1.58 (1.37, 1.83)	0.90 (0.35, 2.34)<br>1.00 (0.98, 1.02)	1.30 (0.69, 2.45)<br>1.01 (0.99, 1.03)	0.895	0.10
	2	CAD only (N=497)	1.60 (1.47, 1.75)			0.861	
		CAD, age (N=493)	1.60 (1.47, 1.75)	0.87 (0.46, 1.64)<br>1.00 (0.99, 1.01)		0.860	0.84
		CAD, service (N=254)	1.50 (1.32, 1.72)		1.30 (0.75, 2.25)<br>1.00 (0.98, 1.01)	0.831	0.41
		CAD, age, service (N=252)	1.52 (1.32, 1.73)	0.74 (0.33, 1.66)<br>1.00 (0.98, 1.02)	1.42 (0.79, 2.55)<br>0.99 (0.98, 1.01)	0.834	0.82
SilicoTB (ILO ≥ 1/0)	1	CAD only (N=497)	1.95 (1.68, 2.26)			0.883	
		CAD, age (N=493)	1.95 (1.68, 2.27)	1.72 (0.47, 6.33)<br>0.99 (0.97, 1.01)		0.884	0.50
		CAD, service (N=254)	1.69 (1.34, 2.14)		1.21 (0.41, 3.60)<br>1.01 (0.99, 1.03)	0.892	0.17
		CAD, age, service (N=252)	1.66 (1.31, 2.10)	0.91 (0.15, 5.72)<br>1.01 (0.98, 1.04)	1.08 (0.32, 3.58)<br>1.01 (0.99, 1.04)	0.897	0.16
	2	CAD only (N=497)	1.68 (1.47, 1.91)			0.839	
		CAD, age (N=493)	1.67 (1.47, 1.91)	1.18 (0.39, 3.54)<br>1.00 (0.98, 1.02)		0.838	0.88
		CAD, service (N=254)	1.60 (1.32, 1.95)		1.20 (0.43, 3.34)<br>1.00 (0.98, 1.02)	0.835	0.81
		CAD, age, service (N=252)	1.60 (1.31, 1.94)	0.84 (0.20, 3.54)<br>1.00 (0.98, 1.03)	1.24 (0.43, 3.58)<br>1.00 (0.98, 1.02)	0.835	0.66

[a] ILO ≥ 1/0 as threshold.
[b] p-value, DeLong's test, comparing the ROC curve of the CAD only model with the indicated model.
AUC = area under the curve, OR = odds ratio, CI = confidence interval.

Figure S1. Chest x-ray reading form

Study # **501** **Miner chest radiology study** **Reader** **1**

1 Quality 1 = Good, all criteria met, 2 = Few defects ① ② ③ ④
3 = Defects but can classify, 4 = Unreadable

2 **Is this CXR AbNormal?** 0 No, 1 Yes ⓪ ①
Stop if No

3 **Silicosis** Profusion 0/0, 0/1 etc ⓪①②③/⓪①②③

PMF? 0 No, 1 Yes ⓪ ①

4 **Intra-thoracic TB?** 0 No, 1 Yes ⓪ ①
Skip to 23

5 **Zones** **(# zones affected by TB) 0 - 6** ① ② ③ ④ ⑤ ⑥

6 **Fibrosis or Fibrocystic disease** 0 No, 1 Yes

Ru	⓪ ①	⓪ ①	Lu
Rm	⓪ ①	⓪ ①	Lm
RL	⓪ ①	⓪ ①	LL

7 **TB Nodules** 1 <1.5mm, 2 1.5-3mm, 3 3-10mm

Ru	①②③	①②③	Lu
Rm	①②③	①②③	Lm
RL	①②③	①②③	LL

8 **Infiltrates** 0 No, 1 Yes
TB non-nodular patchy infiltrates

Ru	⓪ ①	⓪ ①	Lu
Rm	⓪ ①	⓪ ①	Lm
RL	⓪ ①	⓪ ①	LL

9 **Cavities** 0 No, 1 Yes

Ru	⓪ ①	⓪ ①	Lu
Rm	⓪ ①	⓪ ①	Lm
RL	⓪ ①	⓪ ①	LL

10 **Large opacity or mass** 0 No, 1 Yes

Ru	⓪ ①	⓪ ①	Lu
Rm	⓪ ①	⓪ ①	Lm
RL	⓪ ①	⓪ ①	LL

11 **Volume loss** 0 No, 1 Yes

Ru	⓪ ①	⓪ ①	Lu
Rm	⓪ ①	⓪ ①	Lm
RL	⓪ ①	⓪ ①	LL

12 **Bronchiectasis** 0 No, 1 Yes

Ru	⓪ ①	⓪ ①	Lu
Rm	⓪ ①	⓪ ①	Lm
RL	⓪ ①	⓪ ①	LL

13 **Miliary disease** 0 No, 1 Yes ⓪ ①

14 **Apical cap/thickening** 0 No, 1 Yes R ⓪ ① | ⓪ ① L

15 **Tracheal deviation** (mark to which side) R ⓪ ① | ⓪ ① L

16 **Lymphadenopathy** R ⓪ ① | ⓪ ① L

17 **Hilar elevation** R ⓪ ① | ⓪ ① L

18 **Pleural effusion** R ⓪ ① | ⓪ ① L

19 **Pleural thickening** R ⓪ ① | ⓪ ① L

20 **Any other finding consistent with TB?**
Free text:

21 **Any evidence of active PTB?** ⓪ ① ②
0 Nil, 1 Possible, 2 Probable / Definite

22 **Any evidence of previous PTB?** ⓪ ① ②

23 **Listed non-TB, non-silicotic abnormalities** 1 No, 1 Yes ⓪ ①
If yes: 01 Other infection 02 Solid mass
03 Pneumothorax 04 Trauma including fractures ① ② ③ ④ ⑤ ⑥ ⑦
05 Bullae 06 Hyperinflation 07 Cardiomegaly (size or shape)

24 **Unlisted non-TB, non-silicotic abnormalities**
Free text:

Record findings "consistent with" and be rather more sensitive than specific. The default is 0. Use red ink please
